# Supplementary material for: Highly concentrated trehalose induces prohealing senescence-like state in fibroblasts via CDKN1A/p21
Source: Commun Biol. 2023 Jan 6;6:13. doi: 10.1038/s42003-022-04408-3 (PMC9822918; doi:10.1038/s42003-022-04408-3)

Supplemental Information

Highly concentrated trehalose induces prohealing senescence-like state in fibroblasts via CDKN1A/p21

Authors: Jun Muto[1*], Shinji Fukuda[2], Kenji Watanabe[3], Xiuju Dai[1], Teruko Tsuda[1], Takeshi Kiyoi[4], Kenji Kameda[1], Ryosuke Kawakami[5], Hideki Mori[1], Ken Shiraishi[1], Masamoto Murakami[1], Takeshi Imamura[5, 6], Shigeki Higashiyama[7, 8], Yasuhiro Fujisawa[1], Yoichi Mizukami[3], Koji Sayama[1]

Affiliations:
[1]Department of Dermatology, Ehime University Graduate School of Medicine; Toon, Japan.
[2]Department of Biochemistry, School of Dentistry, Aichi Gakuin University; Nagoya, Japan.
[3]Institute of Gene Research, Yamaguchi University Science Research Center; Yamaguchi, Japan.
[4]Department of Pharmacology, School of Medicine, Kanazawa Medical University, Uchinada, Japan.
[5]Department of Molecular Medicine for Pathogenesis, Ehime University Graduate School of Medicine; Toon, Japan
[6]Translational Research Center, Ehime University Hospital; Toon, Japan
[7]Division of Cell Growth and Tumor Regulation, Proteo-Science Center, Ehime University; Toon, Japan.
[8]Department of Molecular and Cellular Biology, Osaka International Cancer Institute; Osaka, Japan.

*Corresponding author. Email: muto.jun.xs@ehime-u.ac.jp

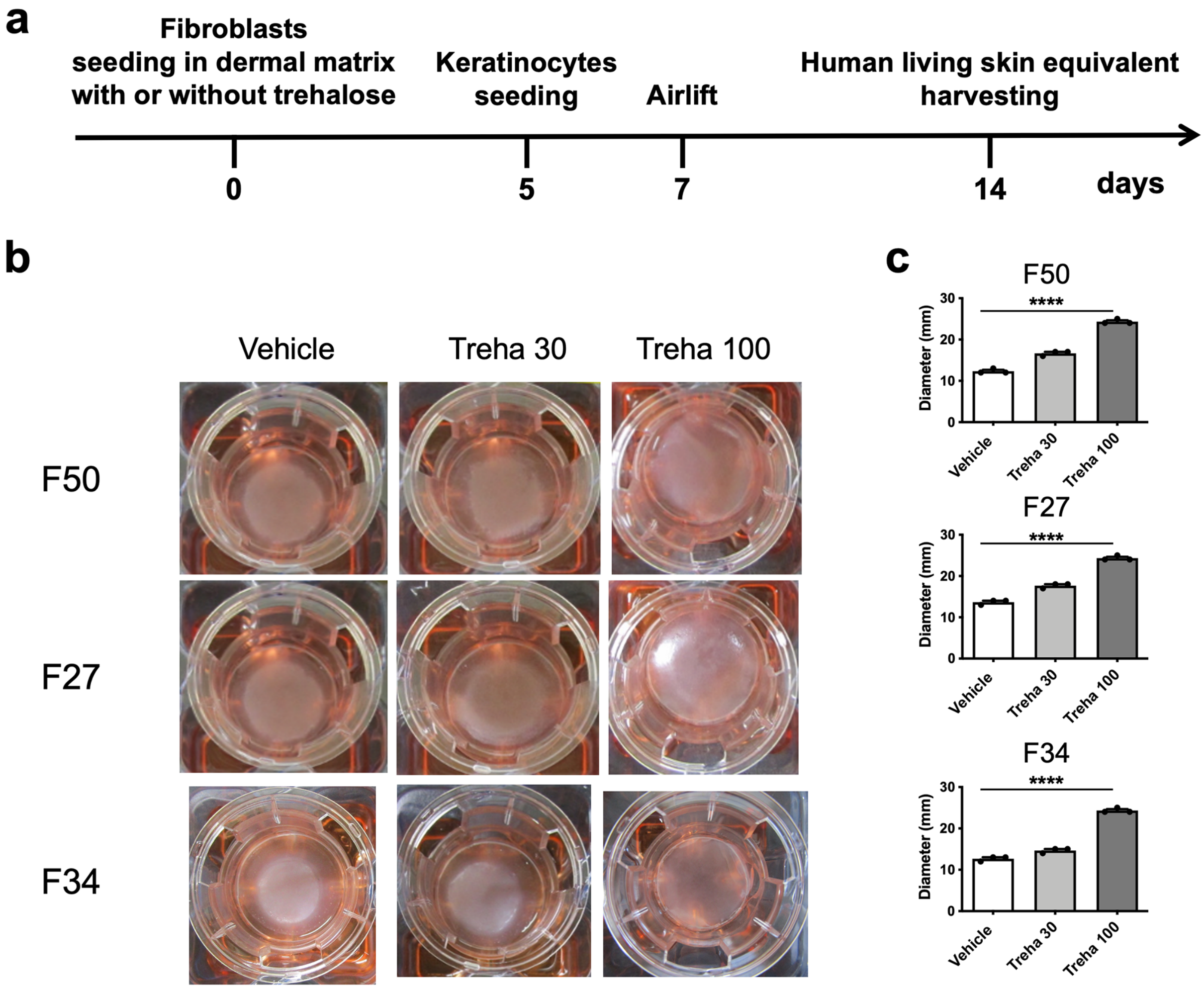

Supplementary Figure 1. Effect of highly concentrated trehalose in the preparation of living skin equivalents. (**a**) A schematic for the preparation of cultured skin equivalents. (**b**) Macroscopic pictures of LSEs with and without trehalose (30 and 100 mg/ml) added in the collagen gel. The gel was prepared in the Transwell-COL with a 24-mm insert in a 6-well culture plate after 1 week of airlifting at 37°C. (**c**) Diameters of LSEs with and without trehalose added in the collagen gel after 1 week of airlifting at 37°C. Data are expressed as means ± SD for three LSEs. ****: $P < 0.0001$ versus vehicle control groups by Student's *t*-test.

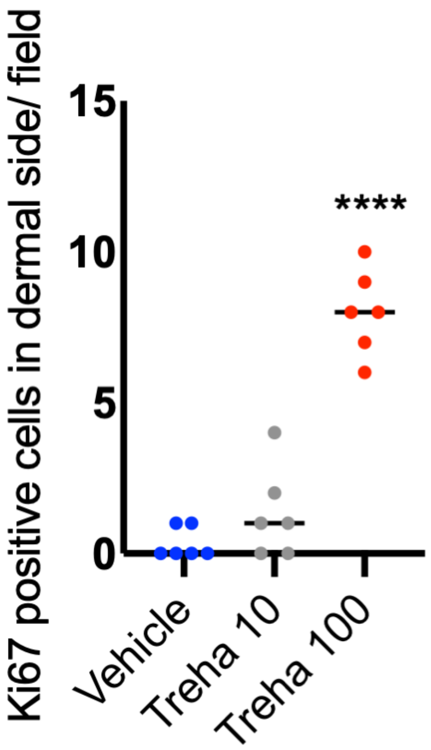

Supplementary Figure 2. Quantification of immunostaining of living skin equivalents prepared with or without trehalose (10 and 100 mg/ml). The numbers of Ki-67 positive cells in the dermal side of LSEs with and without trehalose added in the collagen gel after 1 week of airlifting at 37°C were counted in six randomly selected high-power fields. Data are expressed as means ± SD. ****: $P < 0.0001$ versus vehicle control and trehalose (10 mg/ml) groups by one-way ANOVA.

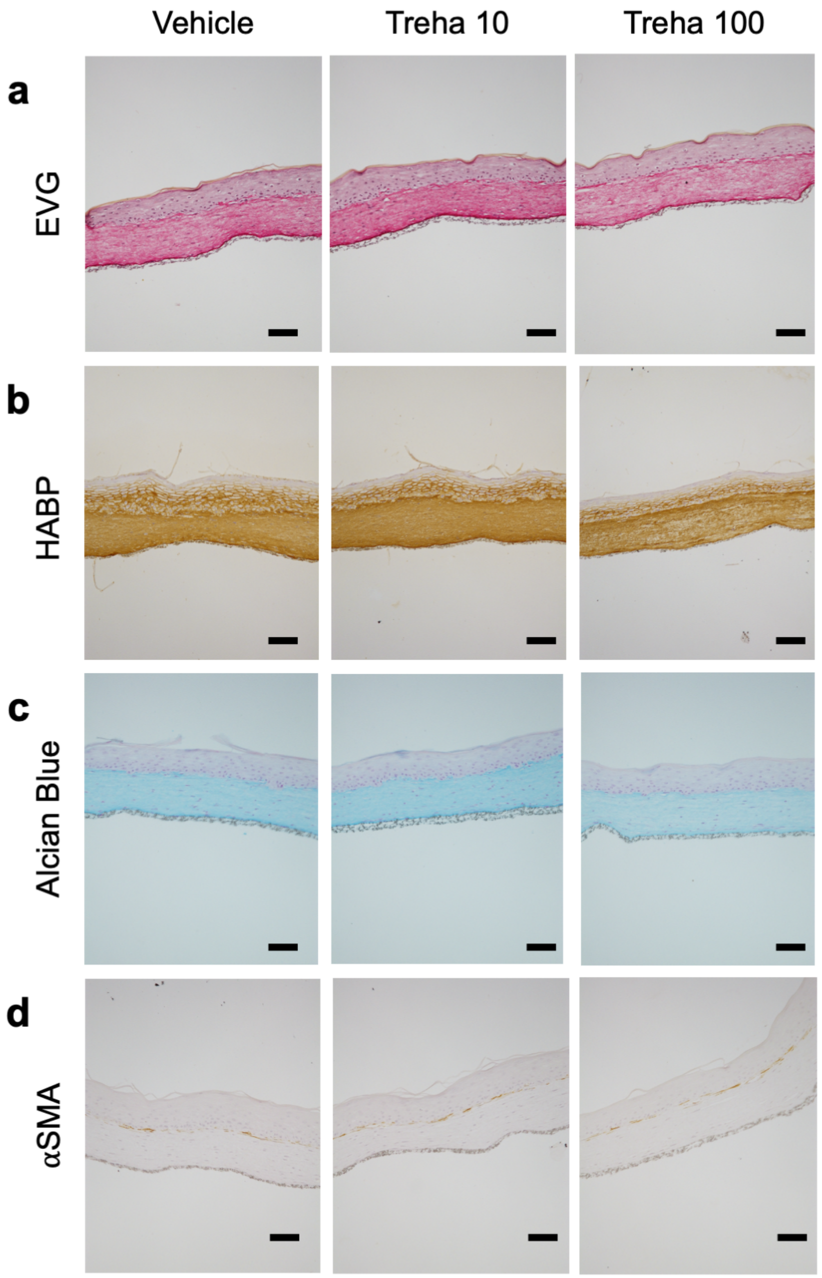

Supplementary Figure 3. Immunostaining of living skin equivalents prepared with or without trehalose (10 and 100 mg/ml). Paraffin-embedded sections of LSEs were sectioned and subjected to immunohistochemistry for Elastica van Gieson staining (**a**), hyaluronan-binding protein (HABP) staining (**b**), Alcian blue staining, pH 2.5 (**c**), α-SMA staining (**d**). Scale bar = 50 µm.

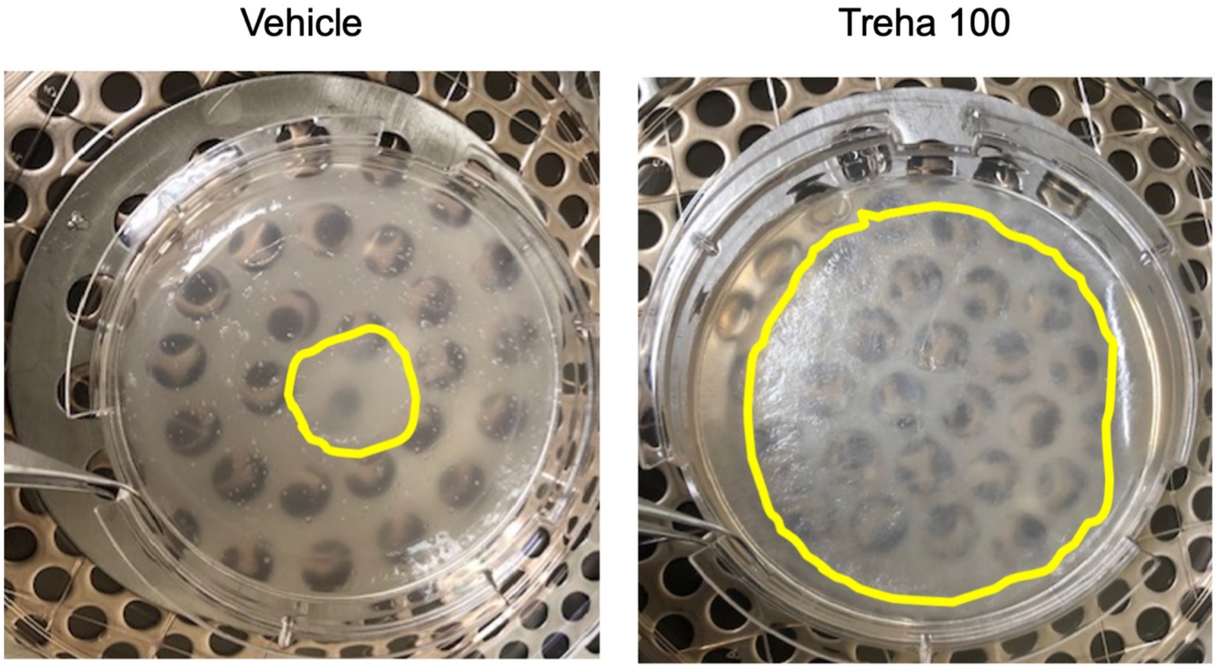

Supplementary Figure 4. Novel effect of trehalose in the preparation of living skin equivalents. Representative picture of LSEs with or without trehalose (100 mg/ml) added in the collagen gel, which was prepared in a 100-mm dish after 2 weeks of airlifting at 37°C. Data are representative of two independent experiments.

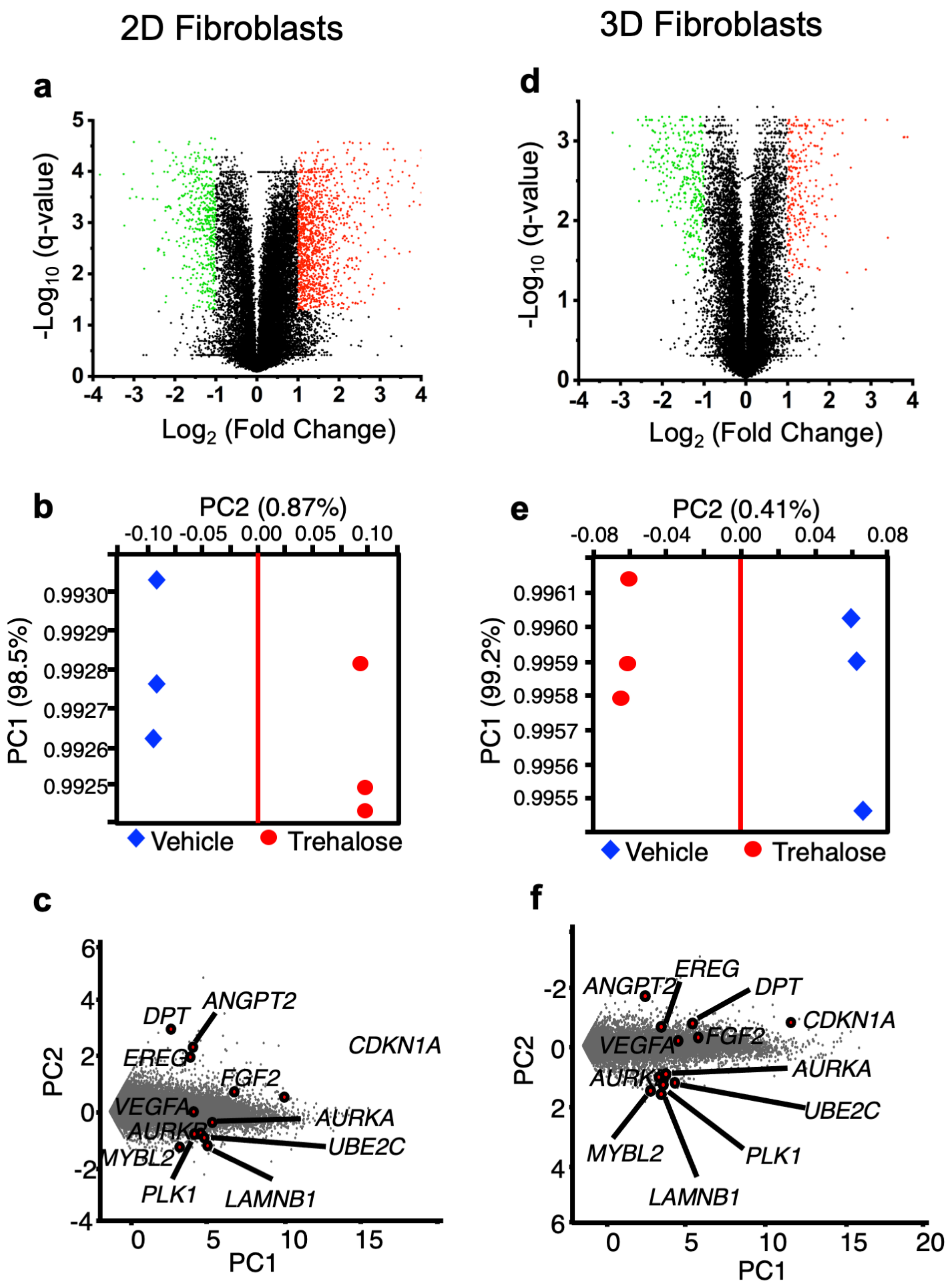

2D Fibroblasts
3D Fibroblasts
a
-Log10 (q-value)
Log2 (Fold Change)
d
-Log10 (q-value)
Log2 (Fold Change)
b
PC2 (0.87%)
PC1 (98.5%)
Vehicle
Trehalose
e
PC2 (0.41%)
PC1 (99.2%)
Vehicle
Trehalose
c
PC2
PC1
DPT
ANGPT2
EREG
CDKN1A
FGF2
VEGFA
AURKA
AURKB
UBE2C
MYBL2
PLK1
LAMNB1
f
PC2
PC1
ANGPT2
EREG
DPT
CDKN1A
FGF2
VEGFA
AURKA
AURKB
UBE2C
MYBL2
PLK1
LAMNB1

80

Supplementary Figure 5. Gene expressions in highly concentrated trehalose-treated 2D and 3D fibroblasts after whole transcriptome analysis by RNA-seq. Volcano plots show that in the presence of trehalose (100 mg/ml) in 2D fibroblasts (**a**) and 3D fibroblasts (**d**), gene expressions were significantly modulated. Red or green rounds indicate genes upregulated by more than 2-fold or downregulated by less than 0.5-fold, respectively, with less than 0.05 of q-values. PCA showing the separation between PC1 and PC2 in 2D fibroblasts (**b**) and 3D fibroblasts (**e**). The factor loadings of PC1 and PC2 of the genes calculated by PCA were plotted. The plotted upper and lower genes were detected in 2D fibroblasts (**c**) or 3D fibroblasts (**f**).

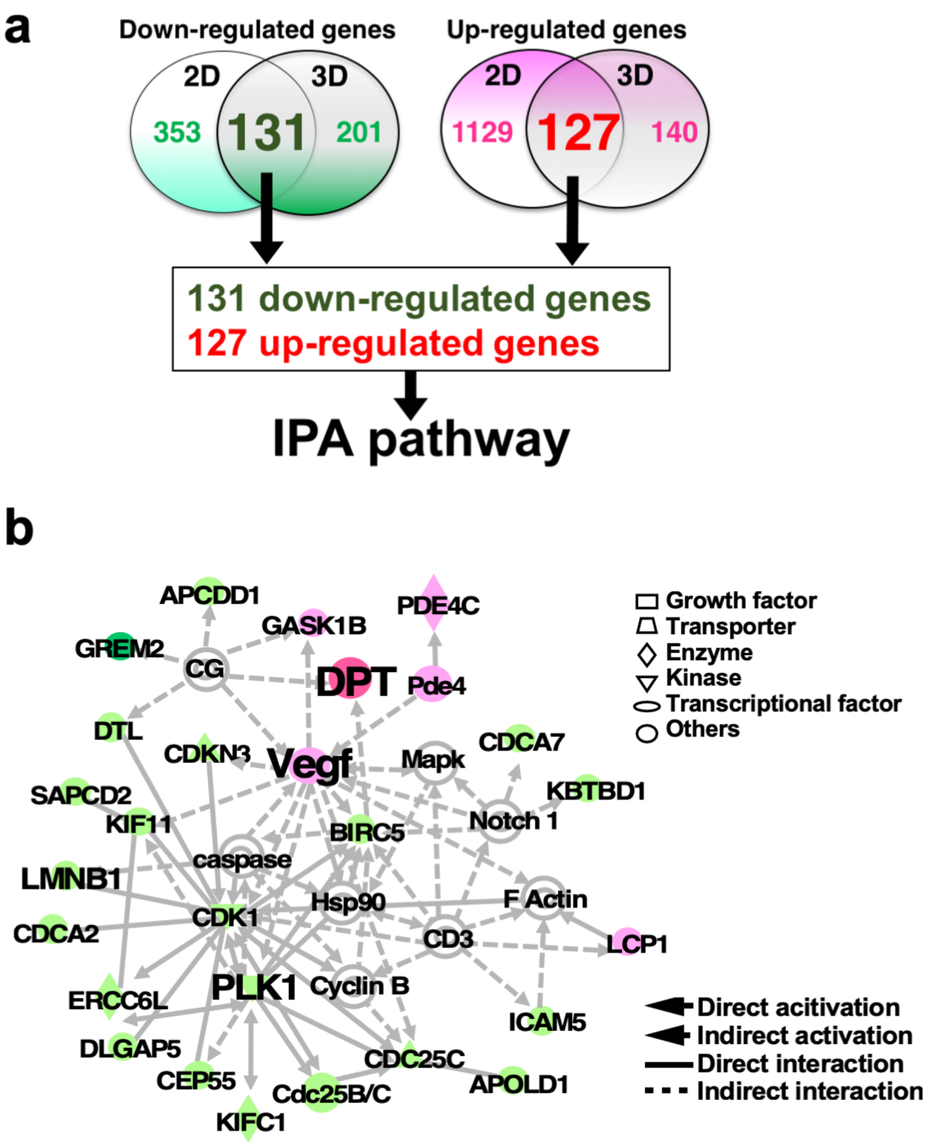

Supplementary Figure 6. Network analysis by IPA pathway using genes modulated by trehalose. (**a**) The Venn diagram demonstrates the number of downregulated and upregulated genes analyzed in Fig. 2. The 131 downregulated genes and 127 upregulated genes were used for an IPA. (**b**) A network detected by IPA demonstrated activation of DPT and Vegf, accompanied by inhibition of PLK1, LMNB1, and CDK1. The red and green shapes demonstrate upregulated and downregulated genes by trehalose, respectively.

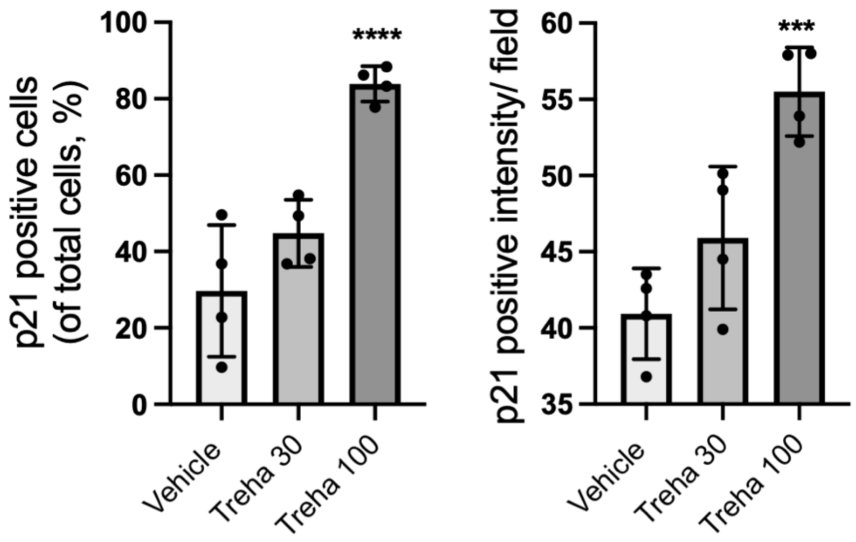

Supplementary Figure 7. Trehalose modulates the expression of p21. Human dermal fibroblasts were treated with trehalose (30 and 100 mg/ml) or vehicle (PBS) for 24 h. The cells were stained with an antibody against p21 and DAPI for nuclei, and both were observed using a fluorescence microscope. In each group, we observed the relative number and intensity of fibroblasts stained by the p21 antibody. Data are means ± SD for four wells, and are representative of three experiments with similar results. ***: $P < 0.001$, ****: $P < 0.0001$ versus the control (vehicle-treated) fibroblasts by one-way ANOVA.

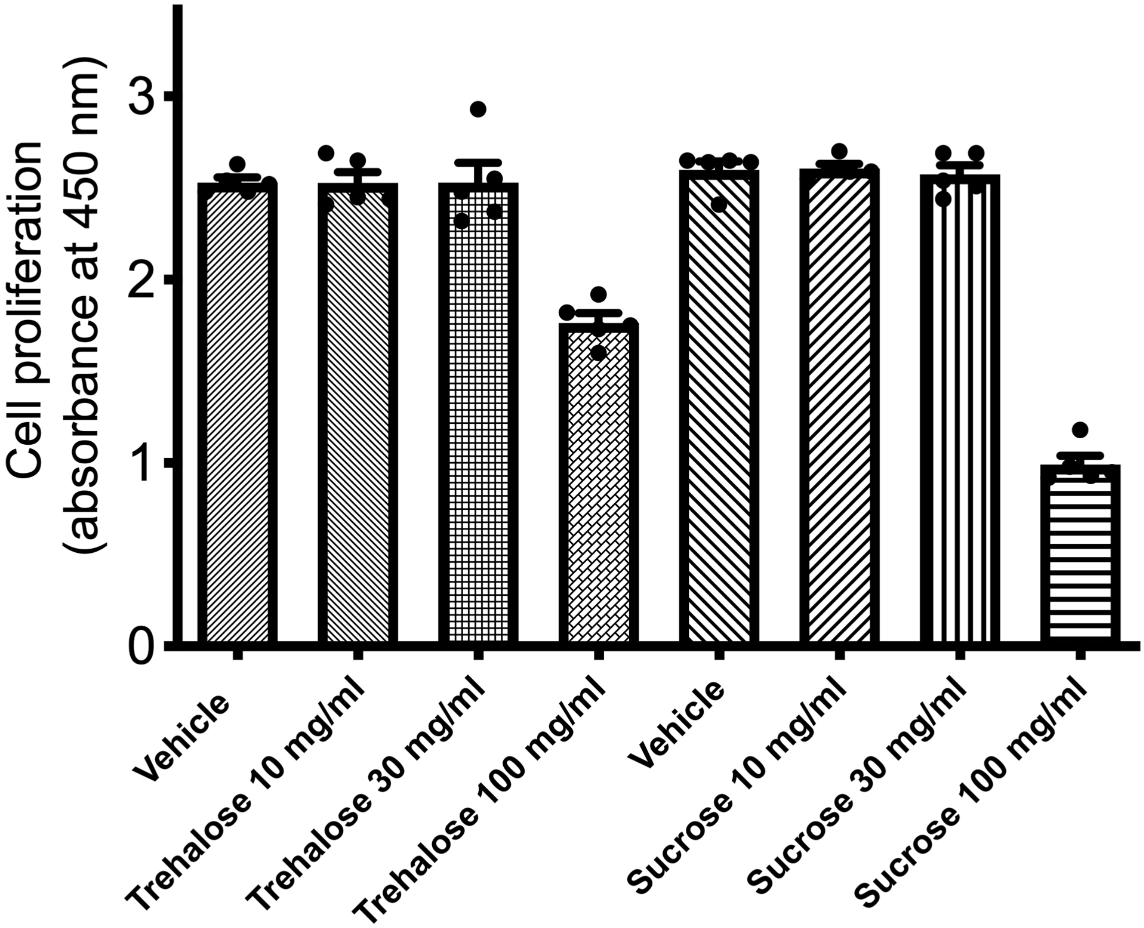

Supplementary Figure 8. Proliferation assay with trehalose-treated human dermal fibroblasts. Proliferation assay with human dermal fibroblasts treated with trehalose (10, 30, and 100 mg/ml) or vehicle for 24 h.

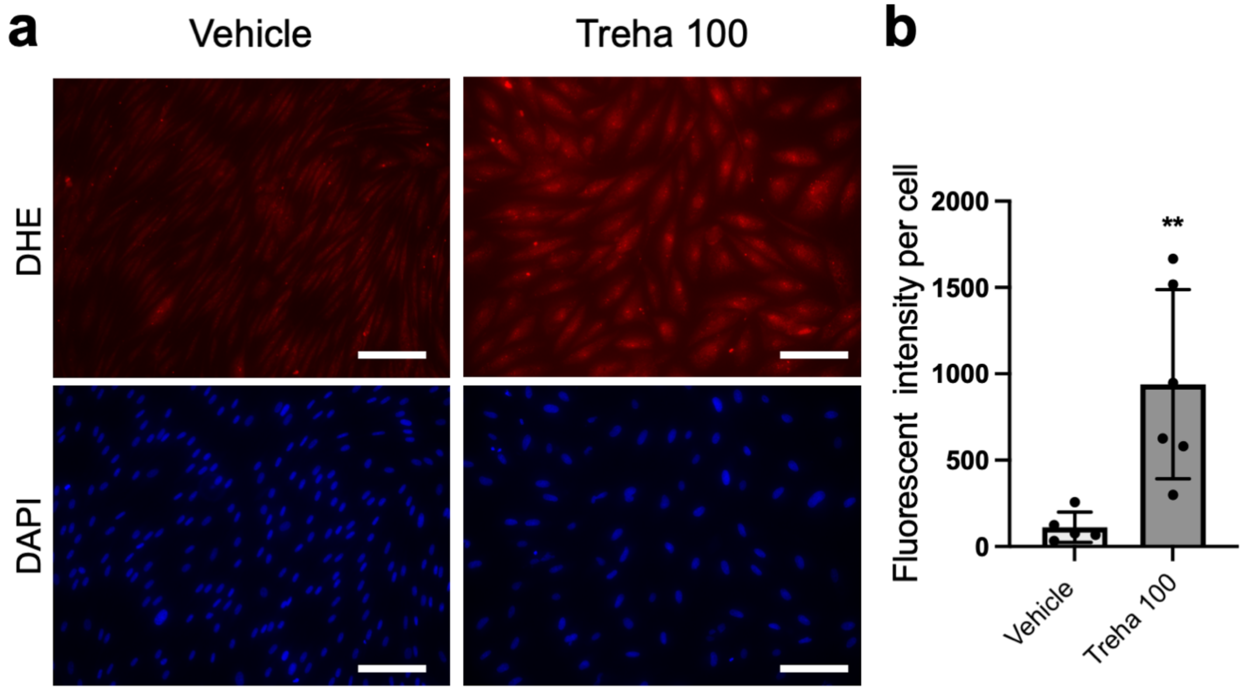

Supplementary Figure 9. Fluorescent images of trehalose-treated fibroblasts stained with DHE. (**a**) Fibroblasts were treated with vehicle or trehalose (100 mg/ml) for 48 h and stained with DAPI to visualize nuclei. Representative fluorescent images of DHE-treated cells were obtained by fluorescence microscopy. (**b**) In each group, we observed the intensity per cell stained with DHE. Data are means ± SD for five wells (vehicle) and six wells (trehalose) and are representative of two experiments with similar results. **: $P < 0.001$ versus the control (vehicle-treated) fibroblasts by Student's *t*-test. Scale bar = 100 μm.

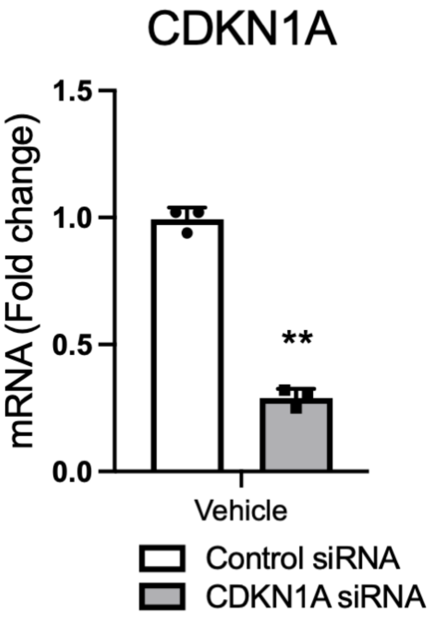

Supplementary Figure 10. CDKN1A is involved in the promotion of wound healing with the dermal substitute containing trehalose-treated fibroblasts. *CDKN1A* mRNA levels were assessed by qPCR after transfection with control or *CDKN1A* siRNA for 24 h. Expression data are shown relative to the control siRNA-treated fibroblasts. Data are expressed as means ± SD. **: $P < 0.01$ versus the control siRNA-treated group of vehicle-treated fibroblasts by Student's *t*-test.

	2D Fibroblast			3D Fibroblast		
Gene Symbol	Log_2 Fold Change	P value	q value	Log_2 Fold Change	P value	q value
CCL26	15.6887127	2.0027E-05	0.00044792	1.705299024	0.000739	0.008949
CCL5	1.068414138	0.45589618	0.45288915	-0.173989996	0.327876	0.492259
CTSB	0.971606011	0.06904278	0.1363981	0.335018077	0.000016	0.001276
CXCL1	1.068140074	0.73874177	0.65627578	-0.874653559	0.002441	0.019497
CXCL5	0.94567579	0.75128719	0.66503826	-0.777055659	0.001055	0.011256
IL1B	1.038811561	0.37390097	0.38579554	-0.000435529	0.998081	0.929793
IL8	0.749017012	0.40241364	0.4091766	-1.863078226	0.000022	0.001431
IL6	1.83106492	0.03268861	0.07368166	0.560710784	0.01188	0.058068
IL6ST	0.768246043	0.00028053	0.00211584	-0.153220532	0.008595	0.046269
IL6R	0.961693449	0.60541119	0.56747153	0.340425901	0.022357	0.091428
IL11	1.38408421	0.00127207	0.00602665	-0.641457641	0.000063	0.002266
GDF15	5.504128467	5.3168E-06	0.00021386	2.204582142	0.000023	0.001467
ICAM3	1.10713058	0.17158772	0.26835683	-0.04654121	0.784125	0.816121
IGFBP2	0.816306772	0.21727315	0.32304459	0.153371435	0.499837	0.607437
IGFBP4	0.676750709	1.1438E-05	0.0003231	-0.096631441	0.019633	0.083265
IGFBP6	0.599951743	2.1271E-05	0.0004627	-0.440933601	0.000426	0.006395
INHBA	0.919449668	0.27440813	0.38579554	0.282262443	0.002374	0.019129
KITLG	1.193926054	0.00022606	0.00185082	0.153844064	0.005932	0.035706
LIF	0.733713608	0.00408197	0.01444751	0.198355932	0.053675	0.173018
MMP1	1.107060554	0.00487661	0.01651	0.917704713	0.000062	0.00226
MMP3	1.286485945	3.9542E-05	0.00063832	2.32521748	<0.000001	0.000541
MMP10	1.466808305	0.00679295	0.02126738	1.567801079	0.000048	0.002069
MMP12	1.614463727	0.01064761	0.03018434	0.47100454	0.058762	0.185098
MMP14	0.555567434	7.3144E-06	0.00025431	0.129684845	0.003357	0.024065
PLAT	2.985791621	9.1255E-06	0.00028893	-0.25587261	0.025534	0.100313
PLAU	0.699589106	2.2605E-05	0.0004765	-0.085737884	0.136164	0.323803
PLAUR	0.804912306	0.00707554	0.02193321	-0.448530887	0.000271	0.004945
SERPINB2	0.935304804	0.55097058	0.52692515	-0.108924632	0.364082	0.492259
TIMP1	0.899312334	0.00046492	0.00295382	-0.13252159	0.01695	0.07489
TNFRSF10C	4.022071381	6.6203E-05	0.00085594	2.191704997	0.000001	0.000567
STC1	0.872968807	0.08588855	0.16310923	0.268688045	0.008082	0.044319
HMGB1	0.749005876	3.46E-05	0.00059345	-0.672302379	0.000018	0.001331
CALR	0.844018275	3.6457E-06	0.00017579	-0.358529449	0.000113	0.003023
CD44	0.506002712	1.0211E-07	5.1976E-05	-0.126432638	0.020103	0.084665
S100A11	1.039862234	0.11871843	0.20483225	0.06647586	0.10953	0.293671
LGALS3BP	0.807693816	0.00822613	0.02470954	-0.307984476	0.016317	0.07301
VCAN	0.720397578	0.00094683	0.00486125	-0.47473911	0.000133	0.003325
TNC	3.665322378	1.0098E-07	5.1976E-05	-0.100713946	0.023494	0.094632
HSPA5	0.749125475	7.657E-06	0.00025961	-0.618822695	0.000002	0.000603
HSP90AB1	0.748200289	2.7946E-06	0.00015423	-0.10722718	0.012148	0.059095
HSPA8	1.358284902	1.1828E-05	0.00032903	0.014429089	0.628248	0.707638
HSPA1A	2.360615298	1.9545E-06	0.00012892	-0.659152755	0.007447	0.041969
HSP90AA1	1.105287269	0.00070951	0.0039587	-0.063762606	0.018879	0.081054
HSP90B1	0.645522626	7.4136E-07	0.00010197	-0.874711901	0.000005	0.000797

Supplementary Table 1. Expression of SASP factor genes induced by highly concentrated trehalose. Analysis of RNA-seq data revealed that several genes associated with trehalose-induced premature senescence were upregulated.

Gene symbol	Assay ID	Gene Name
EREG	Hs00914313_m1	Epiregulin
AREG	Hs00950669_m1	Amphiregulin
GAPDH	Hs02758991_g1	Glyceraldehyde-3-Phosphate Dehydrogenase
ARG2	Hs00982833_m1	Arginase 2
PGF	Hs00182176_m1	Placental Growth Factor
VEGFA	Hs00900055_m1	Vascular Endothelial Growth Factor A
DPT	Hs00355056_m1	Dermapontin
CCL2	Hs00234140_m1	C-C Motif Chemokine Ligand 2
SPP1	Hs00959010_m1	Secreted Phosphoprotein 1
IL1RN	Hs00893626_m1	Interleukin 1 Receptor Antagonist
ANGPT2	Hs00169867_m1	Angiopoietin 2
AURKA	Hs00269212_m1	Aurora Kinase A
AURKB	Hs00177782_m1	Aurora Kinase B
AURKC	Hs00152930_m1	Aurora Kinase C
CDKN1A	Hs00355782_m1	Cyclin Dependent Kinase Inhibitor 1A
PLK1	Hs00983227_m1	Polo Like Kinase 1
LMNB1	Hs01059210_m1	Lamin B1
FGF2	Hs00960934_m1	Fibroblast Growth Factor 2
MYBL2	Hs00942540_m1	MYB Proto-Oncogene Like 2
UBE2C	Hs00153153_m1	Ubiquitin Conjugating Enzyme E2 C
HIF1A	Hs00152153_m1	Hypoxia Inducible Factor 1 Subunit Alpha

Supplementary Table 2. TaqMan primers and probe assay ID (Applied Biosystems) of the genes used in real-time PCR.

Video 1. The morphological alterations of the human dermal fibroblasts after vehicle treatment. Phase contrast microscopy imaging of the fibroblasts cultured with vehicle.

Video 2. The morphological alterations of the human dermal fibroblasts after the trehalose treatment (30 mg/ml). Phase contrast microscopy imaging of the fibroblasts cultured with trehalose.

Video 3. The morphological alterations of the human dermal fibroblasts after the trehalose treatment (100 mg/ml). Phase contrast microscopy imaging of the fibroblasts cultured with trehalose.

Video 4. The morphological alterations of the human dermal fibroblasts after the sucrose treatment (100 mg/ml). Phase contrast microscopy imaging of the fibroblasts cultured with sucrose.

Supplementary blots

<u>Figure 4b blots</u>

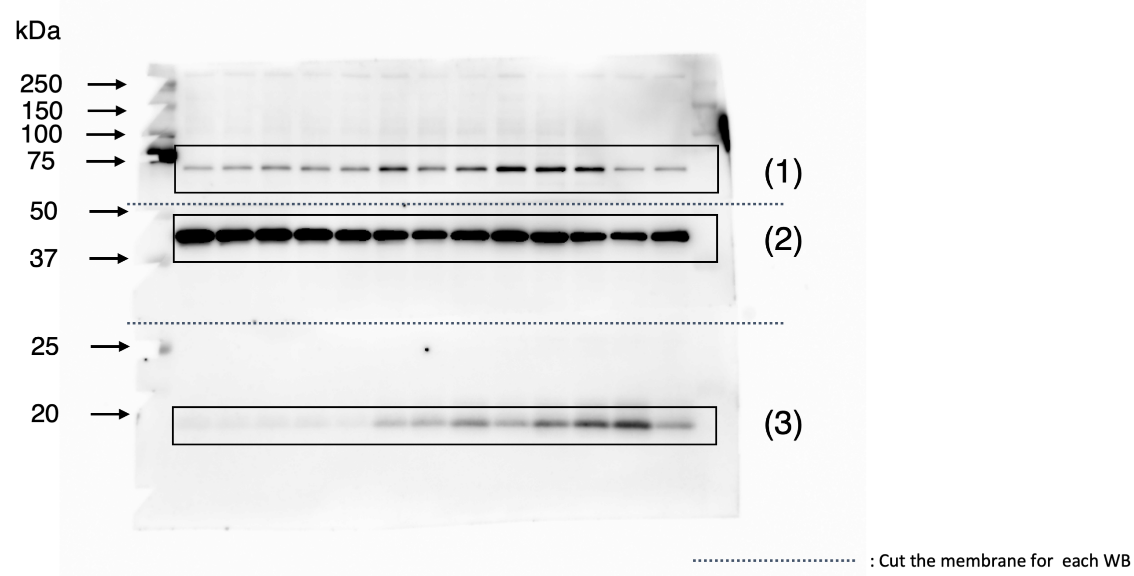

	1st Ab	2and Ab
(1)	LaminB (C-20), #sc-6216 ,Santacruz	Anti goat IgG HRP conjugate, #HAF109, R&D
(2)	β-actin, #ab6276, Abcam	Anti mouse IgG HRP conjugate, #W402B, PROMEGA
(3)	P21(12P1), #29475, Cell Signaling Technology	Anti rabbit HRP conjugate, #W4018, PROMEGA

Figure 5e blots

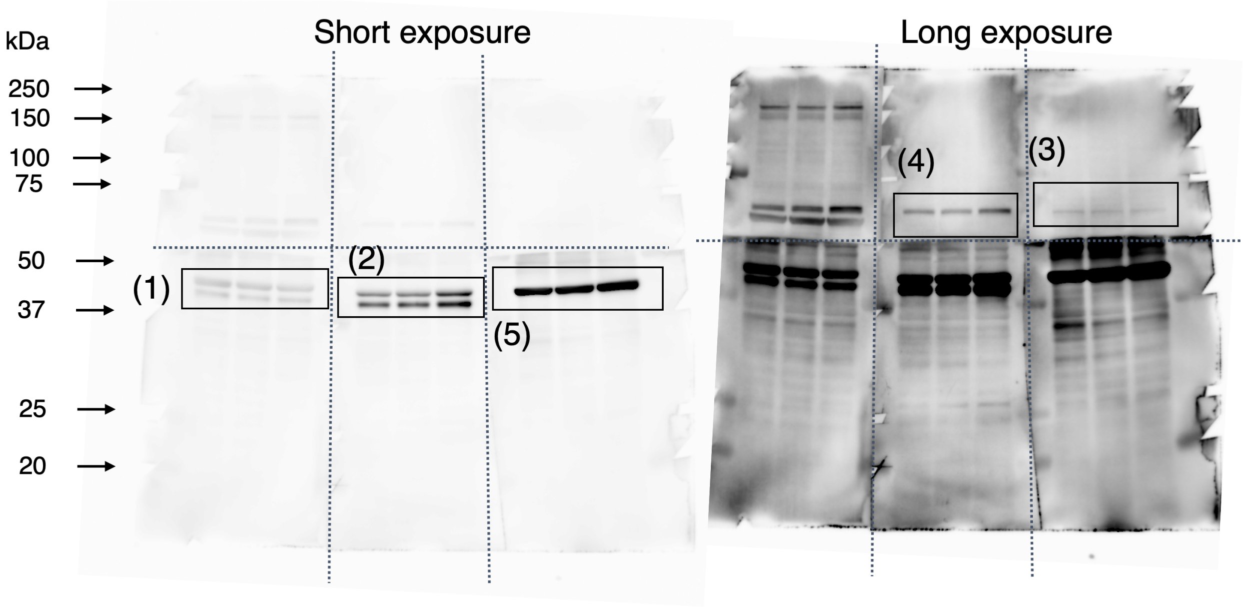

1st Ab

(1) Anti-p44/42 MAPK (Erk1/2), #9102, Cell Signaling Technology

(2) Anti-phospho p44/42 MAPK, #4370S, Cell Signaling Technology

(3) Anti-AKT, #9272, Cell Signaling Technology

(4) Anti-phosphor AKT(Ser 473), #9271, Cell Signaling Technology

(5) Anti-β actin (AC-15), #ab6276, Abcam

2nd Ab

Anti rabbit HRP conjugate, #W4018, PROMEGA

.... : Cut the membrane for each WB

<u>Figure 6c blots</u>

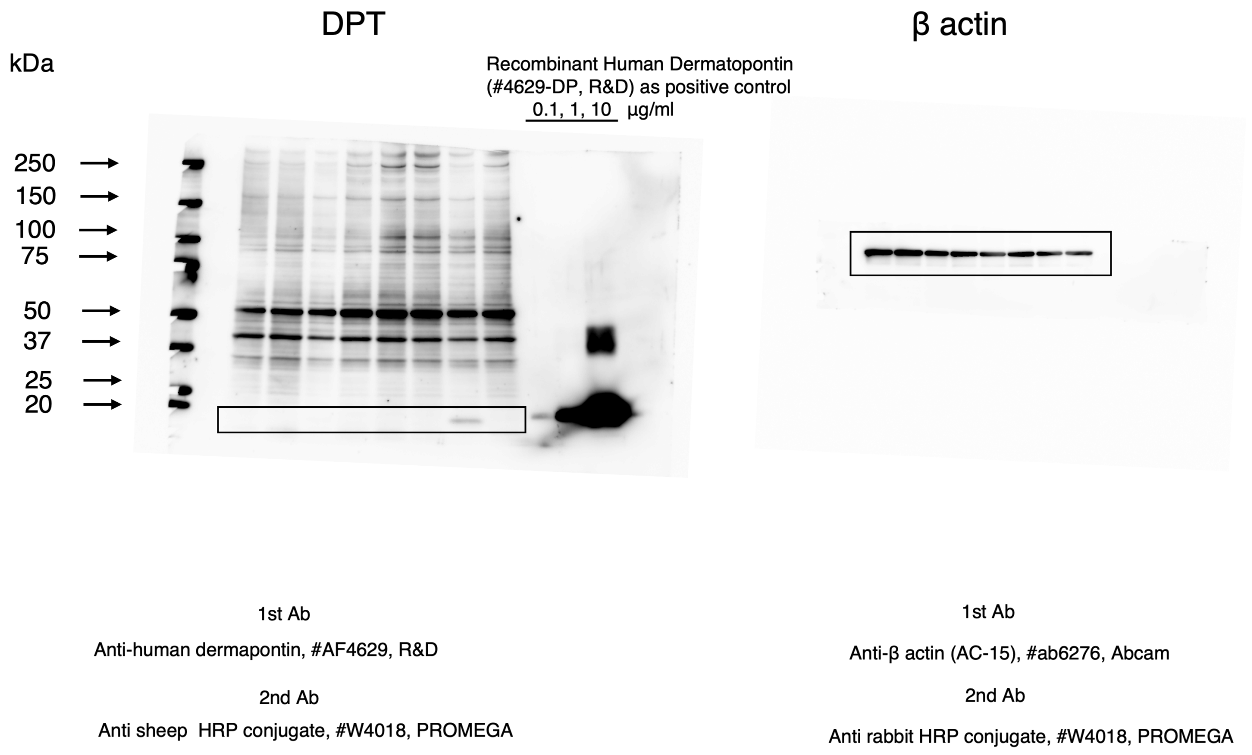

Supplement: Supplementary file 2 — Supplemental Information [file 42003_2022_4408_MOESM2_ESM.pdf]
