# Supplementary material for: Highly concentrated trehalose induces prohealing senescence-like state in fibroblasts via CDKN1A/p21
Source: Commun Biol. 2023 Jan 6;6:13. doi: 10.1038/s42003-022-04408-3 (PMC9822918; doi:10.1038/s42003-022-04408-3)
Supplement: Supplementary file 3 — Description of Additional Supplementary Files [file 42003_2022_4408_MOESM3_ESM.pdf]

Description of Additional Supplementary Files

File name: Supplementary Movie 1
Description: The movie showing the fibroblasts after vehicle treatment.

File name: Supplementary Movie 2
Description: The movie showing the fibroblasts after low dose trehalose treatment.

File name: Supplementary Movie 3
Description: The movie showing the fibroblasts after high dose trehalose treatment.

File name: Supplementary Movie 4
Description: The movie showing the fibroblasts after sucrose treatment.

File name: WB images
Description: Uncropped and unedited blot images.

File name: Figure 1 source data
Description: Source Data of Figure 1.

File name: Figure 4 source data
Description: Source Data of Figure 4.

File name: Figure 5 source data
Description: Source Data of Figure 5.

File name: Figure 6 source data
Description: Source Data of Figure 6.

File name: Figure 7 source data
Description: Source Data of Figure 7.

File name: Figure 8 source data
Description: Source Data of Figure 8.

File name: Figure 9 source data

Description: Source Data of Figure 9.

File name: Supplementary Figure 1 source data

Description: Source Data of Supplementary Figure 1.

File name: Supplementary Figure 2 source data

Description: Source Data of Supplementary Figure 2.

File name: Supplementary Figure 7 source data

Description: Source Data of Supplementary Figure 7.

File name: Supplementary Figure 8 source data

Description: Source Data of Supplementary Figure 8.

File name: Supplementary Figure 9 source data

Description: Source Data of Supplementary Figure 9.

File name: Supplementary Figure 10 source data

Description: Source Data of Supplementary Figure 10.
