# Supplementary material for: Highly concentrated trehalose induces prohealing senescence-like state in fibroblasts via CDKN1A/p21
Source: Commun Biol. 2023 Jan 6;6:13. doi: 10.1038/s42003-022-04408-3 (PMC9822918; doi:10.1038/s42003-022-04408-3)
Supplement: Supplementary file 21 — Reporting Summary [file 42003_2022_4408_MOESM21_ESM.pdf]

nature portfolio

Corresponding author(s): JUN MUTO

Last updated by author(s): Dec 7, 2022

Reporting Summary

Nature Portfolio wishes to improve the reproducibility of the work that we publish. This form provides structure for consistency and transparency in reporting. For further information on Nature Portfolio policies, see our Editorial Policies and the Editorial Policy Checklist.

Statistics

For all statistical analyses, confirm that the following items are present in the figure legend, table legend, main text, or Methods section.

n/a	Confirmed	
☐	☒	The exact sample size (*n*) for each experimental group/condition, given as a discrete number and unit of measurement
☐	☒	A statement on whether measurements were taken from distinct samples or whether the same sample was measured repeatedly
☐	☒	The statistical test(s) used AND whether they are one- or two-sided<br>*Only common tests should be described solely by name; describe more complex techniques in the Methods section.*
☐	☒	A description of all covariates tested
☐	☒	A description of any assumptions or corrections, such as tests of normality and adjustment for multiple comparisons
☐	☒	A full description of the statistical parameters including central tendency (e.g. means) or other basic estimates (e.g. regression coefficient) AND variation (e.g. standard deviation) or associated estimates of uncertainty (e.g. confidence intervals)
☐	☒	For null hypothesis testing, the test statistic (e.g. F, t, r) with confidence intervals, effect sizes, degrees of freedom and P value noted<br>*Give P values as exact values whenever suitable.*
☒	☐	For Bayesian analysis, information on the choice of priors and Markov chain Monte Carlo settings
☒	☐	For hierarchical and complex designs, identification of the appropriate level for tests and full reporting of outcomes
☐	☒	Estimates of effect sizes (e.g. Cohen's *d*, Pearson's *r*), indicating how they were calculated

Our web collection on statistics for biologists contains articles on many of the points above.

Software and code

Policy information about availability of computer code

Data collection	NanoZoomer S210 (Hamamatsu Photonics K. K., Hamamatsu, Japan) and NDP. View 2.9.25 software were used to acquire images.<br>Fluorescence images were taken using fluorescence microscope BZ9000 (Keyence, Osaka, Japan).
Data analysis	Statistical analysis was performed with Prism software (version 9; GraphPad Software, San Diego, CA).<br>Fluorescent signals were quantified using ImageJ (National Institutes of Health).<br>Workbench software (ver.8.01, Qiagen, Hilden, Germany). The pathway was analyzed using Ingenuity Pathway Analysis (Qiagen).<br>Data were also analyzed by JMP pro (SAS Institiute Inc., ver15.0.0).

For manuscripts utilizing custom algorithms or software that are central to the research but not yet described in published literature, software must be made available to editors and reviewers. We strongly encourage code deposition in a community repository (e.g. GitHub). See the Nature Portfolio guidelines for submitting code & software for further information.

Data

Policy information about availability of data

All manuscripts must include a data availability statement. This statement should provide the following information, where applicable:

- Accession codes, unique identifiers, or web links for publicly available datasets
- A description of any restrictions on data availability
- For clinical datasets or third party data, please ensure that the statement adheres to our policy

All raw and processed data will be made available upon request.
High-throughput sequence data are uploaded to GEO under accession number GSE184892. The access code “wlshsqyahbirryt” can be used for it.

Field-specific reporting

Please select the one below that is the best fit for your research. If you are not sure, read the appropriate sections before making your selection.

☒ Life sciences ☐ Behavioural & social sciences ☐ Ecological, evolutionary & environmental sciences

For a reference copy of the document with all sections, see nature.com/documents/nr-reporting-summary-flat.pdf

Life sciences study design

All studies must disclose on these points even when the disclosure is negative.

Sample size	No explicit power analysis was used as the results were relatively consistent between samples.
Data exclusions	Data were not excluded from analysis.
Replication	Information about biological and technical replicates is provided in the respective figure legends. Replicates numbers were decided from experience and practical considerations.
Randomization	No formal randomization method was used.
Blinding	Investigators were not blinded to group allocation.

Reporting for specific materials, systems and methods

We require information from authors about some types of materials, experimental systems and methods used in many studies. Here, indicate whether each material, system or method listed is relevant to your study. If you are not sure if a list item applies to your research, read the appropriate section before selecting a response.

Materials & experimental systems

n/a	Involved in the study	
☐	☒	Antibodies
☒	☐	Eukaryotic cell lines
☒	☐	Palaeontology and archaeology
☐	☒	Animals and other organisms
☒	☐	Human research participants
☒	☐	Clinical data
☒	☐	Dual use research of concern

Methods

n/a	Involved in the study	
☒	☐	ChIP-seq
☐	☒	Flow cytometry
☒	☐	MRI-based neuroimaging

Antibodies

Antibodies used	Anti-CD31 (DIA-310, dianova GmbH, Hamburg, Germany), used at 1:20<br>Anti-α-SMA (n1584, Agilent technologies, Santa Clara, CA), used at 1:1<br>Anti-Ki67 (NCL-Ki67-MM1, LeicaBiosystems, Buffalo Grove, IL), used at 1:100<br>Anti-p21 (#29475, Cell Signaling Technology, Danvers, MA), used at 1:500<br>Alexa Fluor 488-conjugated secondary antibody (###Thermo Fisher Scientific), used at1:200<br>Antibodies for ERK (#9102), phospho-ERK (#4370), AKT (#9272), phospho-AKT (#9271), p21 waf/cipl (12p1) (#29475) (Cell Signaling Technology), used at 1:1000<br>Anti-LaminB (catalog sc-6216) (C-20, Santa Cruz, Dallas, TX), used at 1:1000<br>Anti-DPT (AF4629, R&D systems, Minneapolis, MN), used at 1:1000<br>Anti-beta-actin (#ab6276) (Abcam, Cambridge, UK), used at 1:10000
Validation	These antibodies have been validated for use, as stated on the product page (see website).

Animals and other organisms

Policy information about studies involving animals; ARRIVE guidelines recommended for reporting animal research

Laboratory animals	Ten-week-old male BALB/cAJc1-nu nude mice (CLEA Japan, Tokyo, Japan).
Wild animals	The study did not involve the wild animals.
Field-collected samples	The study did not involve samples collected from the field.

Ethics oversight	All animal procedures performed in this study were reviewed and approved by the Ehime University Institutional Animal Care and Use Committee (05NE46-1).

Note that full information on the approval of the study protocol must also be provided in the manuscript.

Flow Cytometry

Plots

Confirm that:

☒ The axis labels state the marker and fluorochrome used (e.g. CD4-FITC).

☒ The axis scales are clearly visible. Include numbers along axes only for bottom left plot of group (a 'group' is an analysis of identical markers).

☐ All plots are contour plots with outliers or pseudocolor plots.

☒ A numerical value for number of cells or percentage (with statistics) is provided.

Methodology

Sample preparation	The dermis was removed from the epidermis of the LSE cells. The dermis was further digested with collagenase type F and hyaluronidase (both from Sigma-Aldrich) for 60 min followed by fluorescence-activated cell sorting (FACS) analysis with propidium iodide (BioLegend, San Diego, CA) according to the propidium iodide cell cycle staining protocol.
Instrument	Gallios Flow Cytometer (Beckman Coulter, CA) was used for analysis.
Software	Data were analyzed in Flow Jo v. 7.6.5.
Cell population abundance	n/a
Gating strategy	n/a

☐ Tick this box to confirm that a figure exemplifying the gating strategy is provided in the Supplementary Information.
